# Pomegranate peel extract inhibits internalization and replication of the influenza virus: An *in vitro* study

**Published:** 2020

**Authors:** Mohammad-Taghi Moradi, Ali Karimi, Mahmoud Rafieian-kopaei, Mohammad Rabiei-Faradonbeh, Hassan Momtaz

**Affiliations:** 1 *Medical Plants Research Center, Basic Health Sciences Institute, Shahrekord University of Medical Sciences, Shahrekord, Iran*; 2 *Cellular and Molecular Research Center, Basic Health Sciences Institute, Shahrekord University of Medical Sciences, Shahrekord, Iran*; 3 *Department of Pathobiology, Infectious Disease and Public Health, School of Animal and Veterinary Sciences, University of Adelaide, Adelaide, Australia*; 4 *Department of Microbiology, College of Veterinary Medicine, Shahrekord Branch, Islamic Azad University*

**Keywords:** Anti-influenza virus, Pomegranate, Punica granatum L., Mechanisms

## Abstract

**Objective:**

Influenza virus, which is associated with high level of morbidity and mortality, has been recently considered a public health concern; however, the methods of choice to control and treat it are limited. Our previous study showed anti-influenza virus activity of pomegranate peel extract (PPE). In this study, the mechanism through which PPE acts against influenza virus A/Puerto Rico/8/34 (H1N1; PR8) was investigated.

**Materials and Methods:**

Ethyl alcohol extract of pomegranate (*Punica granatum* L.) peel was prepared, and the action mechanism of PPE in inhibiting influenza replication was studied by time-of-drug-addition assay, virucidal activity, RNA replication, hemagglutination inhibition assay, viral mRNA expression, and western blot analysis.

**Results:**

PPE inhibited viral polymerase activity, viral RNA replication, and viral protein expression but could not affect hemagglutination inhibition and virucidal activity. According to time-of-drug-addition assay results, PPE inhibited the virus adsorption and early steps of influenza replication.

**Conclusion:**

This study demonstrated that the antiviral effect of PPE on influenza virus is most probably associated with inhibition of viral adsorption and viral RNA transcription.

## Introduction

As an emerging public health issue, influenza virus is one of the most prevalent causes of respiratory tract infection that leads to high morbidity and mortality rates in humans. Annual vaccination is the main approach to prevent influenza infection. Anti-influenza drugs are also useful to prevent and treat the infection (Hayden, 2006 ▶). Currently, there are three groups of anti-influenza agents to fight influenza infection: 1- Amantadine and rimantadine are matrix protein (M2) ion-channel inhibitors that interfere with intracellular viral uncoating in the host. These are effective only against influenza virus A but have widely acquired resistance to it; 2- neuraminidase (NA) inhibitors such as oseltamivir and zanamivir are widely used to treat seasonal and pandemic influenza virus infections (Jackson et al., 2011 ▶); and 3- cap-dependent endonuclease inhibitor such as baloxavir marboxil (CDC, 2018). However, oseltamivir-resistant H1N1 strains have spread since 2007–2008 (Dapat et al., 2013 ▶; Van Der Vries et al., 2013 ▶). Because of the constant risk of the emergence of antiviral-resistant influenza strains, it is important to consistently seek out drugs that could be used to prevent and treat influenza. As there are few approaches to control and treat this infection, plant extracts including pomegranate peel extract (PPE) can be a treatment of choice. 

Pomegranate (*Punica granatum* L., from family *Punicaceae*) is one of the oldest edible fruits that is cultivated in many countries (Elyatem Kader, 1984 ▶). It is extensively cultivated in Iran, Egypt, Russia, Spain, France, China, Japan, Argentina, USA, and India. Pomegranate is extensively used in the folk medicine of Iran and many other countries. Many studies have indicated the therapeutic efficacy of pomegranate against different types of disorders (Ibrahium, 2010 ▶; Haber et al., 2011 ▶; Ismail et al., 2012 ▶; Colombo et al., 2013 ▶; Asmaa et al., 2015 ▶). 

Although pomegranate peel is sometimes considered an agro-waste, it is indeed a source of different flavonoids such as kaempferol, kaempferolerol-3-O-glucoside, epicatechin, catechin, epigallocatechin-3-gallate, flavan-3-ol, kaempferol-3-O-rhamnoglycoside, luteolin 7-Oglucoside, luteolin, naringin, pelargonidin, prodelphindin, quercetin, and rutin with reported antiviral, antibacterial, anti-inflammatory, antioxidant, and antineoplastic bioactivities (Plumb et al., 2002 ▶; Lansky Newman, 2007 ▶). Our previous study showed that PPE had interesting antiviral activity against Influenza virus in MDCK cells (Moradi et al., 2017 ▶). The aim of this study was to investigate the action mechanism of PPE against Influenza A/PR/8 virus in MDCK cells. 

## Materials and Methods

**Plant collection and extraction**

The pomegranate (*Punica granatum* L.) used in this study was from the *Malas* variety obtained from Shahreza, a central region of Iran. The peel powder was dissolved in 80% ethanol and kept at room temperature for 96 hr. Next, the mixture was filtered and concentrated under nearly vacuum pressure at 40°C in a rotary evaporator. 

**Cell culture and influenza virus propagation**

Influenza virus A/Puerto Rico/8/34 (H1N1; PR8) and Madin Darby Canine Kidney (MDCK) cell line were provided from the Influenza Unit of Pasteur Institute of Iran. MDCK cells were grown in Dulbecco’s Modified Eagle’s Medium (DMEM, Gibco, USA) with 10% fetal bovine serum (FBS, Gibco, USA), 100 U/ml penicillin, and 10 μg/ml streptomycin (Pen/Strep, Gibco, USA) at 37ºC in a humidified incubator with 5% CO_2_.

**Virus titration**

A standard 50% tissue culture infectious dose (TCID_50_) method was applied for virus titration (Kim et al., 2010 ▶). At 90% confluence, MDCK cells were prepared in 96-well plates, the cell culture medium was aspirated and washed twice with phosphate-buffered saline (PBS). Then, 200 μl of 10-fold dilutions of virus in DMEM with 0.5 μg/ml trypsin TPCK was added into the wells and incubated for 2 days. Afterwards, 50 μl of culture medium was taken from each well and transferred to a U-bottomed 96-well plate for hemagglutination assay (WHO, 2011 ▶). TCID50 was calculated by the method of Reed and Muench (Reed and Muench, 1938 ▶). 

**Cytotoxicity assay**

The effect of PPE on the viability of MDCK cells was determined using 3-(4, 5-dimethylthiazol-2-yl)-2, 5-diphenyltetrazoliumbromide (MTT, Sigma, USA) assay according to a previously described method (Mosmann, 1983 ▶) with some modifications. Briefly, when the cell monolayer was confluent, the cells were incubated with 200 µl/well of various concentrations of the extract in 96-well plates for 48 hr. Then, cell monolayers were incubated with 50 μl of 1 mg/ml MTT in PBS at 37°C for 4 hr and treated with 100 μl of acidic isopropanol (0.05 NHCl in absolute isopropanol). Afterwards, the plates were shaken for 15 min, the absorbance was read using a reference filter at 640 nm wavelength using a microplate reader (StataFax2100, USA).

**Time-of-drug-addition assay**

To determine the stage of the viral life cycle that is affected by the PPE, MDCK cells were seeded into 24-well plates and incubated overnight until 90% confluence was achieved. Next, the cells were incubated with 10^4^ TCID50 of the virus for 1 hr at 37ºC, and washed with PBS, and then fresh medium containing TPCK and maximum non-toxic concentration (30 µg/ml) of PPE was added. The PPE was added before the adsorption of the virus (-2 to -1 hr), at adsorption (-1 to 0 hr) and at three time points post adsorption (0-2, 2-4, and 4–8 hr). After 8 hr incubation, supernatants were taken and virus progeny yield was determined using TCID_50_ assay (Matusevich et al., 2015 ▶; Zarubaev et al., 2015 ▶). In brief, when 90% confluence was achieved, MDCK cells were prepared in 96-well plates, the cell culture medium was aspirated and washed two times with PBS; then, 100 μl of a series of 10-fold dilutions was added to the wells and left to incubate for 2 days. After 48-hr incubation, virus replication was investigated by hemagglutination assay (Kim et al., 2010 ▶; Organization, 2011; Matusevich et al., 2015 ▶). TCID_50_ (log10) was calculated by the method of Reed and Muench (Reed and Muench, 1938 ▶).

**Virucidal activity**

Maximum non-toxic dilution (30 µg/ml) of PPE and PBS was incubated with 10^4^ TCID50 of the virus for 1 hr at room temperature. Then, the infectious titer of the virus was determined using TCID_50_ assay (Matusevich et al., 2015 ▶).

**Inhibition of viral mRNA expression and viral RNA synthesis**

Confluent MDCK cells were infected with the virus and treated with different concentrations (30, 10, and 3 µg/ml) of the extract in 12-well plates for 13 hr (He et al., 2011 ▶). Total RNA was extracted from the MDCK cells that were inoculated with either untreated or treated viruses by Trizol (Invitrogen, Waltham, MA, USA) based on the manufacturer’s instructions. The extracted RNA was transcribed into cDNA using RevertAid First Strand cDNA synthesis kit (Thermo Scientific, Lithuania) including an influenza A viral RNA-specific universal primer from Uni12 primers (5’-AGCAAAAGCAGG-3’) to detect viral RNA (vRNA) or oligo-dT primers to detect viral mRNA (vmRNA), RNase inhibitor, 5X Reaction buffer, and dNTP Mix to a final volume of 20 μl. Real-time PCR was conducted to detect the vRNA and vmRNA. The vmRNA and vRNA levels were quantified by a RotorGene Q (Corbett, Qiagen, Germany) containing 1 μl of synthesized cDNA solution, 5 μl of 2X SYBER Green Master Mix (Thermo scientific, Lithuania), and 500 nM of each primer (Table 1) to a final volume of 10 μl. Amplification protocol consisted of denaturation at 95ºC for 10 min followed by 45 cycles at 95ºC for 15 sec, 72ºC for 25 sec and, and 54 or 58ºC for 20 sec. Next, the specificity of the amplified products was confirmed with reference to melting curve analysis. All the reactions were performed in two independent repeats in duplicate. 

**Table 1:** Primers used in real-time RT-PCR

**Gene name**	**Primer sequence**	**Melt temperature (** ^o^ **C)**	**Product length (bp)**
***Nucleocapsid protein (NP)***	F:TGTGTATGGACCTGCCGTAGC R:CCATCCACACCAGTTGACTCTTG	62.1 61.3	158
***Hemagglutinin (HA)***	F:CCTGCTCGAAGACAGCCACAACG R:TTCCCAAGAGCCATCCGGCGA	60.6 58.3	98
***Nonstructural protein (NS1)***	F:CATAATGGATCCAAACACTGTGTC R:CCTCTTAGGGATTTCTGATCTCGG	58.1 60	138
***Matrix protein (M)***	F: GGCAAATGGTACAGGCAATG R: AGCAACGAGAGGATCACTTG	57.7 57.6	144
***GAPDH***	F: GGAGAAAGCTGCCAAATATGACGA R: CGAAGGTGGAAGAGTGGGTGT	61.4 61.9	140

GAPDH: Canis lupus familiaris glyceraldehyde-3-phosphate dehydrogenase

A mixture without cDNA template was used as negative control. The expression of mRNA was normalized against that of the control housekeeping gene (*GAPDH*) and the relative changes in gene expression were calculated using the relative quantitative method (2^-ΔΔCt^). The effects on viral RNA synthesis were calculated by absolute quantification and represented as log10 copy number decrements in treatments.

**Construction of the plasmid standards for quantification**

For quantification of real-time PCR assay, the amplified M2 gene was T/A cloned in pTZ57R/T vector (Invitrogen, San Diego, CA). Vectors were transformed into *Escherichia coli* TOP10F' competent cells using calcium chloride solution and under heat shock (42°C) for 90 sec. To screen recombinant vectors, competent cells were cultured in Luria broth (LB) agar (Merck Co., Germany) containing IPTG (0.1 M), ampicillin (100 µg/ml), and Xgal (20 mg/ml) at 37°C overnight. The white colonies were selected and cultured again in LB agar (containing ampicillin) at 37°C overnight. The recombinant vectors were extracted from bacterial cells by Plasmid Mini Extraction Kit (Bioneer, South Korea) according to the manufacturer’s instructions and PCR was conducted to confirm cloning. The concentrations of extracted plasmids were measured spectrophotometrically using a Nanodrop system (Implen Nano Photometer™, Germany). The plasmid copy number was calculated according to the size of the original plasmid alongside the cloned inserts and the concentration of the construct was calculated by the following formula: 

Number of copies/μl = (6.02 × 10^23^copies × plasmid concentrations (g/μl))/ (Number of bases pairs × 660 daltons/base) (Godornes et al., 2007 ▶).

To plot standard curves, ten-fold serial dilutions of the plasmid were prepared and the M2 gene in plasmids was amplified in triplicate for each standard dilution point. Real time RT-PCR quantification was done and the expression of the gene was measured.

**Hemagglutination inhibition (HI) assay**

The HI assay was used to investigate the effect of the extract on influenza virus hemagglutinin (Abdal Dayem et al., 2015 ▶). Briefly, 50 μl of two-fold serial dilutions of the extract were diluted in PBS containing 0.1% BSA mixed with 50 μl of the virus in PBS containing 0.1% BSA. After 1 hr of incubation, the HI assay was carried out to measure virus titration. Titers were expressed as HA units/50 μl (HAU/50 μl) in comparison with the control treatment (virus without extract).

**Western blot analysis**

MDCK cells in 6-well plates were inoculated with PR8 at 100 TCID_50_ in the presence of crude extract (30 and 15 µg/ml) for 24 hr at 37°C. The culture medium was removed and cells were washed twice with cold PBS and lysed in ice-cold radioimmunoprecipitation assay buffer (6X) containing a protease inhibitor cocktail. Protein concentration was measured by Bradford assay (BioRad), and equal amounts of protein from each sample underwent blotting. 

The protein lysates were mixed with SDS loading buffer (0.125 M Tris-HCl 4%, SDS 20%, glycine 10%, and 2-mercapto-ethanol), boiled for 5 min, separated by 12% sodium dodecyl sulfate polyacrylamide gel electrophoresis (SDS–PAGE) and transferred to polyvinylidene difluoride (PVDF; Bio-Rad, USA) membrane using semi-dry transfer system (Bio-Rad, USA). Viral nucleoprotein (NP), M1 protein, and cellular β-actin protein (loading control) were detected using anti-NP (Thermo Fisher Scientific, USA), anti-M1 (Thermo Fisher Scientific, USA) and β-actin antibodies (Abcam; USA), respectively. The horseradish peroxidase conjugated secondary antibodies (goat anti-rabbit/IgG) were used and the bound antibodies were detected using C-DiGit® Blot Scanner (LI-COR, USA).

**Statistical analysis**

Data analysis was conducted by SPSS software (Ver. 16). Kruskal-Wallis test was used to investigate intergroup differences. A p value<0.05 was considered statistically significant.

## Results

**PPE affects an early stage of virus infection**

**Figure 1:**
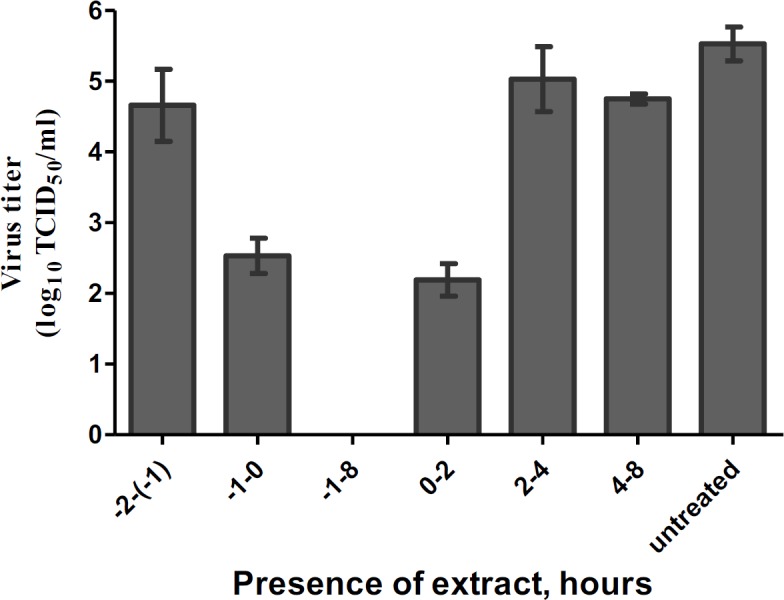
Time-of-addition activity of pomegranate peel extract on influenza virus. Influenza A virus was absorbed to MDCK cells (10^4 ^TCID_50_) for 1 hr. After washing non-absorbed virus, the crude extract was added at its maximum nontoxic concentration (30 µg/ml) to the cell-virus system at the following time points: -2 to -1 hr (before the adsorption of the virus), and -1 to 0 hr (adsorption) as well as at several time points post adsorption: 0–2, 2–4, and 4–8 hr. Virus titer was determined after 8 hr of incubation in MDCK cells using TCID_50_ assay

To determine the stage of viral cycle where the extract exhibited its activity, time-of-drug-addition assays were performed on one cycle of influenza virus replication. The measurements of virus titer showed that virus titer decreased most markedly when the compound was present in the system during the whole test. In addition, the virus titer decreased significantly at time points (-1 hr) to 0 and 0 to 2 hr (Figure 1).

**PPE inhibits viral protein synthesis**

**Figure 2:**
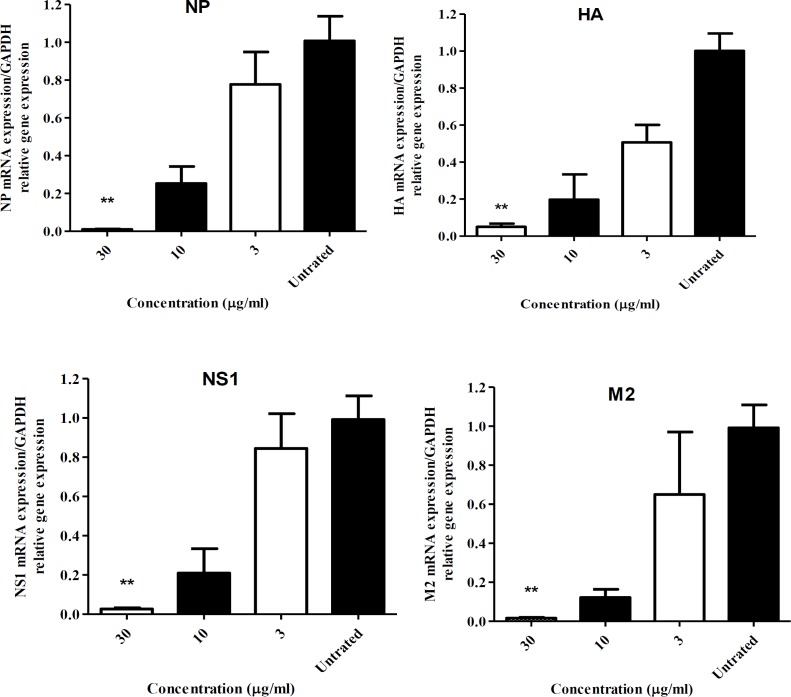
Effects of pomegranate peel extract on the expression of viral mRNA in MDCK cells. Cells were infected with Influenza A virus and treated with different concentrations (30, 10, and 3 µg/ml) of the extract. Total RNA was extracted 13 hr after influenza virus infection and the intracellular levels of mRNA influenza genes (NS1, NP, M1, and HA) were measured. Influenza virus mRNA levels were normalized by *GAPDH*. The data are the mean values of 2 independent repeats in duplicate (mean±SD). **p<0.01 comparison with the virus untreated sample using Kruskal Wallis test

The levels of influenza viral mRNA in the crude extract-treated and untreated infected cells were compared. RNA extraction was conducted at 13 hr after infection with influenza virus, and then the measurements of the intracellular influenza vmRNA (NS1, NP, HA and M2 genes) levels were performed. Quantitative real-time PCR showed a significant and dose dependent decrease of influenza mRNA expression level in the crude extract-treated cells compared with untreated virus infected cells (p<0.05; Figure 2).

**Figure 3:**
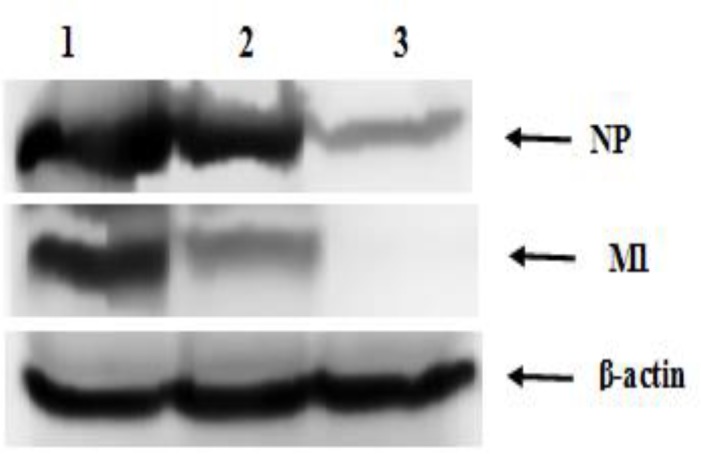
Effects of pomegranate peel extract on protein synthesis in MDCK cells. MDCK cells were infected with Influenza A virus and then incubated with different concentrations (15 and 30 µg/ml) of the extract for 24 hr at 37^o^C. The viral proteins, NP and M1, were detected using their specific primary antibodies and HRP-conjugated secondary antibodies. Cellular β-actin was used as a loading control. Well 1: virus control, well 2: virus + 15 µg/ml crude extract, and well 3: virus + 30 µg/ml crude extract

To investigate viral protein synthesis in the cells after the inoculations of the virus, viral NP and M1 were studied using western blotting. We found that PPE inhibited the viral protein synthesis in a dose-dependent manner (Figure 3). Results indicated that the PPE inhibited the viral protein synthesis. 

**PPE inhibits viral RNA replication**

The synthesis of viral RNA in the cells treated with different concentrations of PPE and untreated cells was compared 13 hr after the inoculation of the virus. The effects of the extract on the levels of viral RNA load were calculated using absolute quantification and shown as log10 copy number decrements in treatments. 

The quantitative analysis of influenza virus M2 gene revealed statistically significant and dose-dependent decrements in viral load when compared to the virus sample (Table 2). 

**PPE does not have virucidal activity**

To investigate the antiviral action mechanism of the extract, a series of tests were conducted to examine its virucidal property and cellular localization. Results showed that virus yield in the extract and control groups was 4.9±0.56 and 5.08±0.59 (log 10-TCID_50_), respectively (p>0.05). This confirmed that the extract did not exert any virucidal effect.

**Table 2:** Effects of PPE on the viral RNA copy number

**Samples**		**Ct (Mean±SD)**	**Log10 Copy Numbers (Mean±SD)**
Virus control (untreated)		19.97±0.283	4.09±0.071
Pomegranate peel treated	30 (µg/ml)	30.05±0.947	1.55±0.238*
	10 (µg/ml)	25.24±0.092	2.77±0.023*
	3 (µg/ml)	22.66±0.0058	3.42±0.001

Cells were infected with Influenza A virus and treated with different concentrations of pomegranate peel extract. Total RNA was extracted 13h after influenza virus infection and the Log10 copy numbers relating to Ct values of intracellular influenza M gene were measured. Data are averages of 2 independent repeats in duplicate. *significant decrease in comparison with the virus untreated sample (p< 0.05).

**PPE does not block the hemagglutination activity**

HI assay was used to determine whether PPE could prevent virus particles binding to cell surface receptors. The HI assay results indicated that pretreatment with the crude extract could not prevent the binding of this virus to RBCs (data not shown). 

## Discussion

*P. granatum* is a frequently used and important herb in folk medicine and its antibacterial, antiparasitic, apoptotic, antifungal, antiproliferative, and antiviral activities have been studied (Kim et al., 2002 ▶; Reddy et al., 2007 ▶; Howell D'souza, 2013 ▶). Few studies have reported the inhibitory effects of *P. granatum* fruit on herpes virus, influenza virus, poxviruses, and human immunodeficiency virus (Haidari et al., 2009 ▶; Howell D'souza, 2013 ▶).  

In this study, the action mechanism of PPE was investigated against influenza A/PR/8 virus in MDCK cells. The results of time-of-drug-addition assay regarding one cycle of influenza virus replication showed that the greatest virus inhibition by PPE was seen at (-1) to 0 hr and 0 to 2 hr after infection. This finding may suggest that PPE inhibits the early step of influenza replication and virus adsorption but does not interfere with the virus binding to the receptors. Besides, our HI assay results indicated that PPE did not inhibit virus-induced hemagglutination. In addition, PPE decreased influenza virus adsorption without influencing HA-mediated virus adsorption. Thus, two possible explanations for the impaired adsorption of influenza virus are; 1) PPE-mediated inactivation of the influenza virus neuraminidase (NA) protein, which cleaves sialic acid in the cell surface and promotes virus internalization (Ohuchi et al., 2006 ▶) and 2) PPE’s ability to induce physical damage to the virus particles, probably the lipid bilayers, which can lead to the loss of their hemifusion capacity. Anti-adsorption effects of this extract deserve further research.

We clearly observed the ability of this extract in reducing the viral polymerase activity and RNA level. Time-of-drug-addition assays also demonstrated that PPE inhibited influenza replication in the early step. It has been reported that before 2.5 hr, the NP and NS1 proteins are preferentially synthesized (Kummer et al., 2014 ▶). The expression of the NP becomes visible 2-4 hr after infection with strong nuclear accumulation (Shapiro et al., 1987 ▶). In influenza virus, the genomic RNAs (vRNAs) are associated with several copies of NPs and a few number of RNA-dependent RNA polymerase complexes consisting of PA, PB1, and PB2, which integrate into ribonucleoprotein complex (RNP) that is necessary for the replication and transcription of the viral RNAs (Sugita et al., 2013 ▶). According to the current study, PPE may decrease the NP level and viral polymerase activity and consequently affect the activities of RNP complexes, leading to inhibition of both transcription and replication of the RNA level. Additional studies, however, should be conducted to identify the precise target of the extract throughout the influenza virus life cycle.

This study demonstrated that *in vitro* antiviral effect of PPE on influenza virus is most probably associated with inhibition of viral adsorption and viral RNA transcription. This extract should be also studied in clinical trials so that it can ultimately be used as a herbal drug against the influenza virus. 
